# Neurons in the thalamic reticular nucleus are selective for diverse and complex visual features

**DOI:** 10.3389/fnint.2012.00118

**Published:** 2012-12-24

**Authors:** Vishal Vaingankar, Cristina Soto-Sanchez, Xin Wang, Friedrich T. Sommer, Judith A. Hirsch

**Affiliations:** ^1^Department of Biological Sciences and Neuroscience Graduate Program, University of Southern CaliforniaLos Angeles, CA, USA; ^2^Computational Neurobiology Laboratory, The Salk Institute for Biological StudiesLa Jolla, CA, USA; ^3^Redwood Center for Theoretical Neuroscience, University of CaliforniaBerkeley, CA, USA

**Keywords:** LGN, TRN, inhibition, receptive field, thalamus

## Abstract

All visual signals the cortex receives are influenced by the perigeniculate sector (PGN) of the thalamic reticular nucleus, which receives input from relay cells in the lateral geniculate and provides feedback inhibition in return. Relay cells have been studied in quantitative depth; they behave in a roughly linear fashion and have receptive fields with a stereotyped center-surround structure. We know far less about reticular neurons. Qualitative studies indicate they simply pool ascending input to generate non-selective gain control. Yet the perigeniculate is complicated; local cells are densely interconnected and fire lengthy bursts. Thus, we employed quantitative methods to explore the perigeniculate using relay cells as controls. By adapting methods of spike-triggered averaging and covariance analysis for bursts, we identified both first and second order features that build reticular receptive fields. The shapes of these spatiotemporal subunits varied widely; no stereotyped pattern emerged. Companion experiments showed that the shape of the first but not second order features could be explained by the overlap of On and Off inputs to a given cell. Moreover, we assessed the predictive power of the receptive field and how much information each component subunit conveyed. Linear-non-linear (LN) models including multiple subunits performed better than those made with just one; further each subunit encoded different visual information. Model performance for reticular cells was always lesser than for relay cells, however, indicating that reticular cells process inputs non-linearly. All told, our results suggest that the perigeniculate encodes diverse visual features to selectively modulate activity transmitted downstream.

## Introduction

One often thinks of cortex as the earliest site where thalamic relay cells drive postsynaptic targets, but this is not the case. En route to cortex, thalamic afferents contact the perigeniculate sector (PGN) of the thalamic reticular nucleus (Bickford et al., 2008), which projects back to the lateral geniculate nucleus (LGN) in turn (Cucchiaro et al., 1991; Uhlrich et al., 1991; Wang et al., 2001). Unlike most brain structures, the PGN is formed entirely by inhibitory cells (Fitzpatrick et al., 1984). These cells communicate with both electrical (Landisman et al., 2002) and chemical synapses (Montero and Singer, 1984), and fire long-lasting bursts of spikes (Burke and Sefton, 1966; Domich et al., 1986; Huguenard and Prince, 1992). Thus, all information that travels to the cortex is influenced by an intricate inhibitory feedback loop.

How do the response properties of neurons in the PGN compare with those of the LGN and cortex? In cat, most cortical cells that receive input from relay cells inherit key properties of the receptive field such as segregated On and Off subregions (Hubel and Wiesel, 1962; Montero and Singer, 2005). Further, like relay cells (Mante et al., 2008), these cortical neurons can be simulated by simple computational models (Carandini et al., 2005). In essence, knowledge of the spatiotemporal receptive field not only describes preferences for visual features but provides a basis to predict responses to novel stimuli.

Many investigators have reported that neurons in the cat's PGN are often binocular and have overlapping On and Off responses (Sanderson, 1971; Cleland and Levick, 1972; Dubin and Cleland, 1977; Ahlsen and Lindstrom, 1982; Uhlrich et al., 1991; Funke and Eysel, 1998). These qualitative studies left an impression that reticular neurons simply pool feed-forward input and provide non-selective inhibition that is modulated by attention (McAlonan et al., 2008). However, later experiments that explored spatial and temporal frequency tuning suggested a greater level of selectivity (So and Shapley, 1981; Xue et al., 1988); in addition, responses in the somatosensory (Hartings et al., 2000) and auditory (Simm et al., 1990) divisions of the reticular nucleus are complex. Further, the projections to and from the PGN are topographic (Fitzgibbon, 2002), a pattern echoed in the auditory (Kimura et al., 2007) and somatosensory divisions of the reticular nucleus (Pinault, 2004; Lam and Sherman, 2007).

Hence we were motivated to explore feature selectivity in the reticular nucleus quantitatively and re-examine the view that the PGN provides a non-selective form of gain control. We adapted techniques (Schwartz et al., 2006) of spike-triggered averaging and covariance analysis to estimate receptive fields that we subsequently employed to build computational models. We used these to determine how well the receptive field predicts neural response and how much information it encodes. Our results suggest the PGN uses complex non-linear mechanisms to encode higher order features of the stimulus and provide selective feedback to the LGN. This result is all the more interesting given recent evidence that second order visual features guide bottom–up processes that direct attention to particular regions of the visual scene (Frey et al., 2007).

## Methods

### Preparation

Adult cats (1.5–3.5 kg) were first anesthetized with a dose of propofol and sufentanil (20 mg/kg + 1.5 μg/kg, i.v.) that was reduced to (5 + 1.5 μg/kg/h, i.v.) for maintenance. The depth of anesthesia was monitored by the EKG, EEG and the absence of an autonomic response; body temperature was held near 37°C. After surgical procedures were complete, the animal was paralyzed with vecuronium bromide (0.2 mg/kg/h, i.v.) and ventilated artificially; expired CO_2_ and blood oxygenation were monitored throughout the experiment. Pupils were dilated with 1% atropine sulphate and the nictitating membranes retracted with 10% phenylephrine. The eyes were refracted and fitted with contact lenses to focus on a tangent screen on which the positions of the area centralis and the optic disk of each eye were marked. A craniotomy centered on Horsley–Clark coordinates A 6.5 and L 8.5 gave access to LGN. All procedures were in accordance with the guidelines of the National Institute of Health and the Institutional Animal Care and Use Committee of the University of Southern California.

### Mulitelectrode recordings

We first used a tungsten electrode to locate the LGN to guide placement of a multielectrode array (Eckhorn and Thomas, 1993). The array was made of 7 independently movable quartz-platinum electrodes (3–4 MΩ, 80 μm) arranged in either a concentric or linear configuration, spaced 305 μm apart. These were lowered into the brain through a glass guide tube (tip diameter 1.1 mm, length 11 mm) whose tip rested approximately 5 mm above the LGN, and, hence, several mm above the PGN (Xue et al., 1988). The angle of the multielectrode was adjusted (25–30° anterior–posterior and 5° medial-lateral) so that the electrode travelled through the same retinotopic position in the thalamus (Yeh et al., 2003). Signals from all electrodes were amplified, filtered, and then stored on a computer running Spike2 software (Cambridge Electronic Design, Cambridge, UK), which we used to extract action potentials for subsequent analysis.

### Visual stimulus

The visual stimulus was displayed on a monochrome CRT (refresh rate 140–144 Hz) using a stimulus generator (ViSaGe, Cambridge Research Design, Ltd., Cambridge, UK). We used two types of stimuli. One was spatiotemporal Gaussian white noise, for which the stimulus intensity of each square in the stimulus grid was sampled from a Gaussian distribution (33% RMS contrast). The stimulus sequence ranged between 4,096 and 16,384 frames, each frame was displayed for 20 ms and repeated 4–16 times. The second stimulus, sparse noise (Jones and Palmer, 1987; Hirsch et al., 1998a), consisted of individually flashed bright and dark squares 29 ms in pseudorandom order, 16 times on a 16 × 16 or 32 × 32 stimulus grid, pixel size 1° or 0.5°, respectively; stimulus contrast was 50% in some trials and 100% in others.

### Localizing the PGN during and after experiments

**Figure 1:**
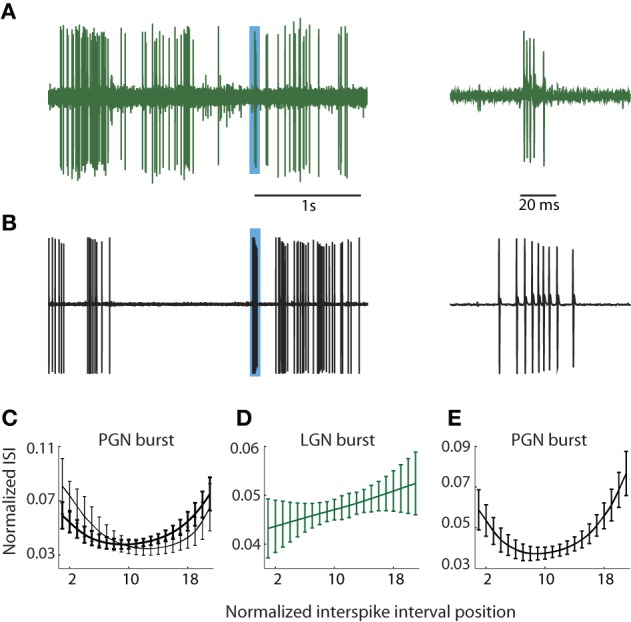
**PGN and LGN burst classification. (A,B)** Sample traces recorded from one cell in the LGN **(A)** and another in the PGN **(B)** depicting tonic and burst spikes, *left*; the regions shaded blue indicate bursts that are shown at an expanded timescale at *right*. Bursts in the LGN are composed of decelerating spike trains whereas bursts in PGN comprise accelerating and decelerating spike trains. **(C)** ISI distributions from two cluster means of bursts obtained from seven randomly selected cells in the PGN; the interval between successive spikes first shortened and then increased to form a U-shaped curve. **(D)** The cluster mean for the sole cluster formed for bursts in the LGN; the ISI grew longer with each successive spike. **(E)** U-shaped cluster mean obtained after pooling events (detected with criteria that identify bursts in the PGN) from seven cells each in the LGN and PGN; all spikes within the cluster came from the PGN.

It had been common to localize the PGN *in vivo* (during the course of the experiment) using qualitative impressions of visual response properties such as binocularity and/or overlapping On and Off responses e.g., Uhlrich et al. (1991). To remove the bias that such assumptions might introduce, we chose a method based on the differences in the structures of bursts of action potentials fired by relay cells and reticular neurons in the PGN. Specifically, relay cells fire brief, decelerating bursts (Burke and Sefton, 1966; Lu et al., 1992) and see Figure 1A (right) whereas reticular neurons fire long bursts in which spike rate first accelerates and then decelerates (Burke and Sefton, 1966; Domich et al., 1986; Huguenard and Prince, 1992; Kim et al., 1997), Figure 1B (right).

During and after experiments, we confirmed that burst pattern accurately localized the recording site using several methods. First, after recording from a putative reticular cell, we lowered the electrode to confirm a sequence of alternating preference for the contralateral and ipsilateral eyes as we moved through the primary (A, A1, and C) layers of the LGN. The A layers were typically encountered at a depth of less than 1 mm from a putative reticular cell, though there was some variation due to the angle of the electrode. Second, at the end of most experiments, we perfused the animal and made histological confirmation of the recording site by tracing the electrode tracks and/or electrolytic lesions. In all cases, the anatomy corroborated physiological assessment. Third, we quantified the differences between bursts in the LGN and PGN to confirm that they provide a signature of recording site, as below.

### Classifying bursts in the LGN and PGN

#### Burst detection

We used the standard criteria to detect bursts in the LGN (Lu et al., 1992; Reinagel et al., 1999). Bursts were defined as two or more spikes, each spaced ≤4 ms apart following ≥100 ms of silence, Figure 1A; bursts rarely lasted more than 10 ms. To detect bursts in the PGN, we adapted metrics similar to those published by Domich et al. (1986) for the reticular nucleus. The criteria were ≥5 spikes within 70 ms, spaced ≤30 ms apart following ≥70 ms of silence; bursts were terminated when the interspike interval exceeded 30 ms, Figure 1B. Thus defined, typical bursts had 5–17 spikes. Typical bursts lasted between 70 and 100 ms.

#### Clustering burst patterns

We wished to establish a metric that would separate bursts in the PGN from those in the LGN. Previous work in the LGN has shown that bursts can be discriminated from tonic spikes based on clusters formed in plots of the interval between one spike and the next vs. the interval between one spike and the previous (Reinagel et al., 1999). Such a metric is not suited for the PGN, however, since the intervals between all but the innermost spikes in a burst are similar to those for tonic spikes. One might also think it possible to separate reliably records from LGN and PGN based on burst length, by measuring the width of the autocorrelogram. We measured the width of autocorrelograms (at 5% of peak height) for spike trains recorded from 10 cells in each structure; the values for the PGN, 19.7 ± 7.7 ms, were wider than for LGN, 6.75 ± 3 ms, (mean ± standard deviation). However, variations in spike rate can alter the widths of these plots for individual cells, so we hesitated to use this measure for purposes of burst classification.

Thus, to establish a quantitative measure to distinguish relay from reticular cells, we focused on differences in the distribution of spike times within bursts. We formed representations of bursts in PGN, Figure 1C, and LGN, Figure 1D, by plotting the interval between successive spikes (ISI). These curves were upsampled using a shape preserving spline interpolating function (Matlab), a process that allowed us to preserve the structure of the ISI distribution while holding the number of points (21) for each burst constant regardless of the number of component spikes. Thus, each burst could be represented as a point in a 21 dimensional space where each ISI was a separate dimension. Next, we used an unsupervised method to cluster bursts detected from groups of neurons in the PGN and/or LGN based on the shape of the ISI [Klustakwik; (Harris et al., 2000)]. The U-shaped distribution of ISIs that is typical of the PGN is depicted in Figure 1C, which plots the means of two clusters formed from bursts fired by 7 randomly chosen reticular cells. By contrast, the ISIs of bursts fired by relay cells grew longer with time, Figure 1D. To confirm that U-shape ISIs are unique to reticular cells, we applied the burst criteria developed for PGN to recordings from the LGN (this test was unilateral since criteria for LGN could select components of bursts in reticular cells). We then pooled these events from the LGN with those obtained from the PGN and formed clusters from this mixture (PGN, *n* = 7; LGN, *n* = 7). This analysis revealed a unique U-shaped cluster mean that corresponded to bursts unique to neurons in the PGN, Figure 1E.

### Recovering linear filters, forming STA and STC subunits from responses to visual noise

#### Gaussian noise

In order to recover the stimulus features (namely subunits, or filters) to which neurons in the PGN are sensitive, we used standard methods to compute the spike-triggered stimulus ensemble for intervals of 0–120 ms stimulus preceding each spike, see Schwartz et al. (2006) for review. The spatiotemporal average of the ensemble formed one subunit, the spike-triggered average (STA). To recover features represented by second order statistics of the spike-triggered stimulus ensemble, we used spike-triggered covariance (STC) analysis (Schwartz et al., 2006). This method computes the eigenvalues and eigenvectors of the covariance matrix (mean removed) of the spike-triggered stimulus ensemble. In order to assess the significance of the subunits recovered with STC analysis, we performed nested bootstrapping to generate surrogate data in which correlations between stimulus and response were removed (Schwartz et al., 2006). Specifically, we shuffled the spike train randomly in time with respect to the stimulus, as is often done in cross-correlation studies, and then performed the same analysis described above (this control left the statistics of the spike train intact as it removed only the temporal relationship between the stimulus and response). By repeating this procedure a thousand times, we were able to estimate a 99% confidence interval of the null hypothesis (that the filters are not significant). If the eigenvalue of a real subunit recovered from STC analysis fell outside the confidence interval, it was considered significant. Thus we recovered both STA and STC subunits that, in aggregate, described the neural spatiotemporal receptive field. We used the same methods to analyze control responses from the LGN.

#### Sparse noise

We formed separate maps for responses to On vs. Off stimuli. Specifically, we formed an STA_ON_ by taking a spatiotemporal average of only the bright stimulus preceding each spike and an STA_OFF_ using the analogous procedure for the dark stimuli. To determine the extent to which On and Off responses overlapped, we calculated the normalized dot product of STA_ON_ and STA_OFF_.

### Adapting spike-triggered methods to the statistics of bursting neurons

**Figure 2:**
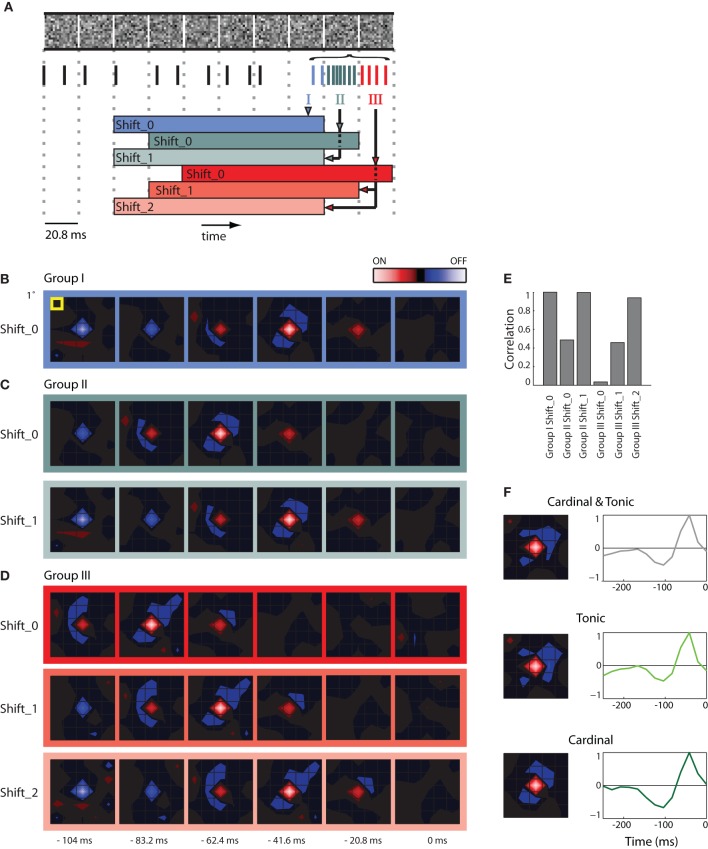
**STAs made from burst and tonic spikes. (A)** Schematic diagram of a sequence of stimulus frames shown above spikes recorded at the same time; dashed vertical lines help align the different components of the figure with the timing of the stimulus. Some spikes formed a burst; these are color coded according to whether they coincided with the first (Group I, blue), second (Group II, green), and third (Group III, red) frame of the stimulus displayed during the burst. The colored horizontal bars, labeled Shift_0, 1, or 2 beneath the spike train delimit the time window used to compute various STAs made for spikes in Groups, I, II, and III. **(B)** STA made with Group I spikes for the interval indicated by the blue bar labeled Shift_0, the peak of the STA emerged 41.6 ms prior to the neural response. **(C)** STAs made from Group II spikes for the stimulus frame indicated by the green bars labeled Shift_0 and Shift_1 (the latter was shifted forward in time by 1 frame-length). Note that both STAs are similar to that illustrated for Group 1, Shift _0 **(B)**, except that the peak of the STA for Group II, Shift_0 is delayed by one frame length. **(D)** Similar results for STAs computed for Group III spikes. **(E)** A bar plot of the correlation strength among STAs made by taking the dot product of STA for Group1 at Shift_0 with the remaining STAs. The strongest correlation between different pairs occurs when the temporal advance, or shift, of time window used to compute the STA compensates for the delay between the cardinal and later spikes in the bursts. **(F)** (*left*) STAs generated using the mixture of cardinal and tonic spikes (*top*), tonic spikes (*middle*), and only cardinal spikes (*bottom*); (*right*) time course of the peak of the STA. As indicated by the colored scale bar in panel **(B)**, red indicates bright stimuli and blue dark stimuli for all spatial maps and the lightness of each color corresponds to stimulus intensity. For all receptive fields, the grid spacing was 1° of visual angle [yellow box, panel **(B)**]. The receptive field was 1.5° from the area centralis.

**Figure 3:**
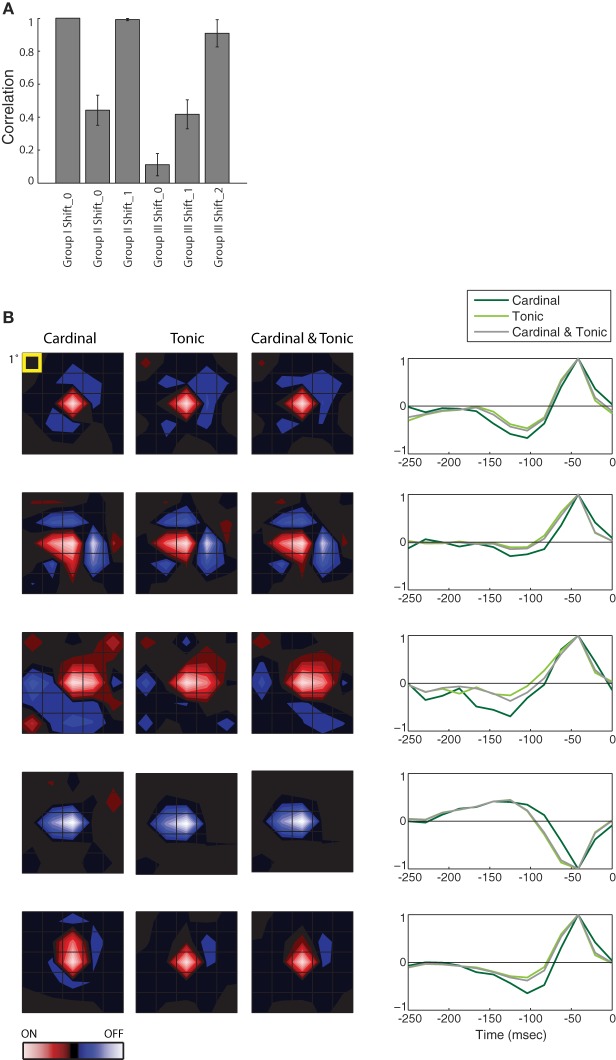
**Population analysis of STAs made from burst and tonic spikes. (A)** Bar plot of the correlation between the STA for Group I at Shift_0 with itself and the various remaining STAs, as labeled, for 10 cells in the PGN. The height of each bar is the population mean and the error bars span ± one standard deviation. **(B)** (left) Each row of maps shows three different STAs, generated either from cardinal spikes, tonic spikes, or a mixture of cardinal and tonic spikes, ordered from left to right as labeled. (right) The time course of the response for the peak pixel of each STA in the same row. Each row illustrates data from a different cell. For comparison, the top row shows results for the same cell as in Figure 2, conventions as in Figure 2. From top to bottom, the eccentricities of the cells were 1.5°, 11.5°, 4°, 4°, and 8.4°.

It is important to note that spike-triggered averaging, like other techniques that use reverse correlation to estimate receptive fields, typically involves the assumption that each impulse is independently driven by the stimulus (Pillow and Simoncelli, 2003). However, as above, reticular neurons generate stereotyped accelerating-decelerating bursts (Burke and Sefton, 1966; Domich et al., 1986) that last far longer than the brief decelerating bursts generated by relay cells (Jahnsen and Llinas, 1984; Lu et al., 1992; Reinagel et al., 1999). Further, work *in vitro* and *in vivo* has shown that this firing pattern is generated by the specialized intrinsic membrane currents that can be triggered by brief depolarizations (Huguenard and Prince, 1992; Contreras et al., 1993). Thus, before completing our analyses, it was necessary to find out whether intrinsic bursts in the PGN might confound our results (see Figures 2 and 3 for a further illustration of the population of cells included in this analysis).

Here we illustrate how we addressed the issue. First, we formed STAs of the stimulus ensemble. The stimulus we used most frequently was Gaussian noise. Each frame of the stimulus consists of a sequence of checkerboards made of squares whose luminance values were drawn from a Gaussian distribution. Then we asked if an excitatory visual stimulus in one frame might account for all spikes in a burst that continued through successive stimulus frames. Our approach was to determine if the spatial maps (STAs) assembled from later spikes in the burst matched the map made from the cardinal spikes, simply shifted back in time, as illustrated in a schematic, Figure 2A [the maps are shown as contour plots (Matlab, contourf)]. The stimulus is depicted in the top row; the dotted vertical lines mark the duration of each frame, 20.8 ms. The spikes in the train recorded during the stimulus are color coded; tonic (non-burst) action potentials are black and burst spikes are shaded according to the frame during which they occurred, as follows. The initial spikes of the burst (Group I, the cardinal action potential as well as others that occurred while the same frame was displayed) are blue; spikes generated during the second frame (Group II) are green; impulses fired during the third frame (Group III) are red. The family of colored horizontal bars at bottom illustrates the compensatory time shifts we made, which we will discuss shortly.

We first illustrate STAs made by reverse correlating spikes in Group I to six frames of the stimulus, blue bordered images, Figure 2B (the label Shift_0 simply means the STA was not shifted in time with respect to the stimulus). The rightmost map was made by averaging the stimuli displayed when the burst was triggered (0 ms) and is followed with STAs made from frames flashed at progressively earlier times before the burst began (*t* = −20.8, −41.6, and −62.4 ms etc.). This time series shows that the receptive field emerged in the second frame, peaked in third and ultimately reversed polarity in the fifth and sixth frames and had a strong On subregion (red) and weaker Off (blue) components.

We then formed similar STAs for spikes from Groups II (Figure 2C, top) and III (Figure 2D, top). In practice, for every burst, we used only one spike in each of the Groups to form the STA so that the contributions from events in each frame were the same. The maps for Groups II and III resemble those for Group I, but shifted backward in time by one frame or two frames, respectively: the shading of the boxes bordering the maps corresponds to the color codes used in the depiction of the shifting process in Figure 2A, bottom. This similarity is consistent with the idea that all the spikes in the burst were driven by the same stimulus. If this were true, then advancing the STAs constructed from Group II by one frame (Shift_1) or from Group III by two frames (Shift_2) should yield an STA that matches that made by Group I. This was the case, as can be seen by comparing the images displayed in Figures 2B–D; specifically, compare the STAs for Group 1, Shift_0, Group II_Shift_1, and Group III_Shift_2.

To quantify the similarity of the STAs made from the different Groups (I, II, and III) at different temporal displacements (Shift_0, _1, and _2), we calculated the dot products between the STAs made for Group I with those made for Groups I, II, and III. The resulting correlations for the six combinations are plotted in Figure 2E. The maximal possible correlation was, of course, between Group I Shift_0 with itself. As predicted from visual inspection of Figures 2B–D, the correlations approached the maximum for Group II when its STA was advanced by one frame (Shift_1) and for Group III with an advance of two frames (Shift_2); compare the three tall bars in Figure 2E. In other words, the STAs made from Groups I, II, and III were essentially indistinguishable, when latency differences were taken into account. Taken together, the analysis supports the idea that the stimulus that precedes the cardinal spike initiates the remaining spikes in that burst. This view is consistent with work in the LGN (Alitto et al., 2005).

Thus, to avoid temporal blurring that correlations between spikes would cause, we excluded later spikes in the bursts from the data used to generate receptive fields. An example STA made from a mixture of tonic spikes and the first (cardinal) spike of each burst is shown in Figure 2F as both a spatial map (top left) and the time course of response measured for the peak pixel (top right). Corresponding plots made only from tonic spikes or only from cardinal spikes are shown at the middle and bottom panels of Figure 2F. All plots are roughly similar.

The results described in Figure 2 hold for the population, the correlation values for different Groups at different Shifts that were plotted for a single cell in Figure 2E are shown for a population of 10 cells in Figure 3A. Only the correlations between STAs at the appropriate time shifts approach 1 (i.e., the three tall bars in Figure 3A); the slight differences are insignificant. Similarly, the STAs and time-courses made from a mixture of tonic and cardinal spikes, or from only tonic or only cardinal spikes are similar, as shown in a plot that compares the results for the cell illustrated in Figure 2 (top row) with plots for four additional cells; Figure 3B. These results recall past work in the LGN that found that the spatial structure of the receptive fields (mapped with binary white noise) for burst and tonic spikes were similar; with the difference being a small disparity in strengths of response (Alitto et al., 2005).

### Estimating and optimizing parameters for linear-non-linear (LN) models

We used a standard linear-non-linear (LN) model (Simoncelli et al., 2004; Carandini et al., 2005) to predict the responses of individual cells in the PGN (and LGN controls), given the stimulus. We evaluated the performance of each model by determining how well it could predict the data that were not used to fit the model, cross-validation (Hastie et al., 2001). Thus, we divided the recordings for each cell in two parts, one used to train the model and the other to test it.

The linear stage of the model was made using linear filters (STA or STC subunits) derived from spike-triggered-analysis. The output of the linear stage of the model was the dot product between the linear filters for a given cell and the stimulus. This signal, often termed the filter output (Rust et al., 2005; Schwartz et al., 2006), captures the similarity between the stimulus and the filter. Here the values of the filter outputs were normalized so that values of 1 or -1 indicate maximum spatiotemporal similarity for stimuli of the same or reverse sign.

The non-linear stage of the model maps the filter output to the neural firing rate. To estimate this mapping function (the spiking non-linearity), we generated a peri-stimulus time histogram (PSTH) by averaging the spike trains evoked by multiple repeats of the same noise sequence. The response non-linearity was estimated by taking the averaged spike count (from the PSTH) for each instance in time in which the visual stimulus evoked the same instantaneous filter output, following procedures developed by others (Chichilnisky, 2001). In practice, the range of values (normalized to the absolute global maximum for the entire stimulus period) of the filter output, 1 to −1, was binned. The bin size was optimized by cross-validation as follows. We successively increased the number of bins in the model and assessed performance for each case. This procedure was halted when an increase in the number of bins reduced rather than improved performance; typically the optimal number of bins was similar across cells (10–12 bins). By optimizing the parameters that maximized the performance of the model, it was possible to avoid the problem of over-fitting.

So far we have described a model that comprises only one linear filter, in other words, a 1D model. For cells with significant STC subunits, we also built (2D) models to explore the combined influence of the STA paired with a significant STC subunit on the neural response (for cells with more than one STC subunit, we made a separate 2D model for each STA/STC pair). For these 2D models, it was necessary to estimate a joint response non-linearity that mapped the outputs of the two subunits to the strength of neural response.

### Assessing model performance

#### Explained variance

The ability of the model to predict the neural response was assessed in two separate ways. First, we estimated the percentage of stimulus-dependent variance in neural response that the model predicted. This metric, the explained variance, is biased by limitations in the length the stimulus sequence and number of repeated trials (David and Gallant, 2005). To address the issue of bias, we adopted a recent method (Haefner and Cumming, 2009) that corrects for two potential confounds; namely, the uncertainty due to the finite amount of data (*N*σ) available for cross-validation as well as the number of free parameters (*n*) in the model. The equation for this modified version of traditional explained variance is written in Equation (1):

(1) $$\gamma [\nu_{0}]=1-\frac{\sum_{i\text{=1}}^{N}{\left(\frac{{\bar{d}}_{i}-m_{i}}{\sigma }\right)}^{\text{2}}-\frac{N_{\sigma }\left(N-n\right)}{N_{\sigma }-\text{2}}}{\sum_{i\text{=1}}^{N}{\left(\frac{{\bar{d}}_{i}-\bar{d}}{\sigma }\right)}^{\text{2}}-\frac{N_{\sigma }\left(N-\text{1}\right)}{N_{\sigma }-\text{2}}}$$

(2) $$\sigma^{\text{2}}\text{=1}/\left(RN\left(R-\text{1}\right)\right)\sum_{i\text{=1}}^{N}\sum_{j\text{=1}}^{R}{\left(d_{ij}-{\bar{d}}_{i}\right)}^{\text{2}}$$

where *d*_*ij*_: neuron's response measured in response to stimulus of length *N* which is repeated R times; ${\bar{d}}_{i}$: neuron's response averaged across stimulus repeats (i.e., the PSTH); *m*_*i*_: model response; *N*σ: *N*(*R*−1); *n*: number of free parameters in the model.

The second, and more straightforward, method estimates the explained variance without accounting for the biases due to finite data or number of free parameters in the model (Sahani and Linden, 2003). Both methods gave similar estimates of model performance, as long as there were sufficient data for cross-validation (when bias due to data limitation was negligible (*N*_σ_ → infinity, noise free).

#### Information theory

We measured the mutual information between filter outputs and actual spike trains to determine how much information the different subunits provided about the neural response. The mutual information (in bit/spike) was estimated as the Kullback–Leibler divergence between the distribution of filter outputs during the entire stimulus period (prior stimulus distribution or unconditioned filter output) and the distribution of filter outputs that preceded spikes (spike conditional distribution or filter output conditioned on the neural response) (Aguera Y Arcas et al., 2003; Fairhall et al., 2006), Equation (3).

(3) $$I_{\text{one spike}}^{\left(K\right)}\left(f_{1},\;f_{2},\;\ldots ,\;f_{K}\right)=\int d^{K}sP\left(s_{1},\;\ldots ,\;s_{K}|\text{spike at }t\right){\text{log}}_{2}\times \left[\frac{P(s_{1},\;\ldots ,\;s_{K}|\text{ spike at }t)}{P(s_{1},\;\ldots ,\;s_{K})}\right]$$

where s_*K*_ represents the K projections (filter outputs) of the stimulus on filters *f*_*K*_, *P*(*s*_*K*_|spike) is the spike conditional distribution and *P*(*s*_*K*_) is the prior stimulus distribution. Similarly the amount of information accounted for by the STA subunit was given by Equation (4) and that by the subspace spanning the joint STA + STC_*K*_ subunits described by Equation (5).

(4) $${\text{I}}^{\left(\text{STA}\right)}=P(s_{\text{STA}}|\text{ spike}){\text{log}}_{\text{2}}\left[\frac{\text{P}(s_{\text{STA}}|\text{ spike})}{\text{P}(s_{\text{STA}})}\right]$$

(5) $${\text{I}}^{\left(\text{STA},\;{\text{ STC}}_{K}\right)}=P(s_{\text{STA}},\;s_{{\text{STC}}_{\text{K}}}|\text{ spike})\times \log_{2}\text{​}\left[\frac{P(s_{\text{STA}},\;s_{{\text{STC}}_{\text{K}}}|\text{ spike})}{P(s_{\text{STA}},\;s_{{\text{STC}}_{\text{K}}})}\right]$$

where *s*_STA_ and *s*_STC_K__are the outputs of the STA and STC_K_ subunits, respectively.

Note that each significant subunit provides information about the neural response only if the prior, *P*(*s*_*K*_), and the spike conditional distributions, *P*(*s*_*K*_| spike), differ. The precision with which the information can be estimated for each of these subunits can change as a function of the bin size used to sample the distributions. Hence we estimated the information for a range of bin sizes, calculated as a multiple of the standard deviation (σ) of the prior stimulus distribution (bin size ranged from 0.01 σ to 1σ). The smallest bin size that can be used, however, is limited by the amount of data that are available (Panzeri et al., 2007). To account for the bias caused by finite sampling of Ps_STA_|spike and P(s_STA_), we also estimated the information carried by the most non-significant subunit, I^(STC_nonsig_)^ (Fairhall et al., 2006). Non-significant subunits, virtually by definition, contain no information about the stimulus. However, due to the bias, the estimate for even the most non-significant subunit is non-zero. Thus, we subtracted this amount of information from the total information encoded by the 1D model, I^(STA)^.

For the 2D model, the bias was again estimated as the difference between I^(STA)^ and paired information I^(STASTC_nonsig_)^ for the most non-significant subunit (Fairhall et al., 2006). After removing the biases, the estimates of information for both models were stable for bin sizes from 0.15σ to 0.4σ. The final information estimate was taken as the mean of the values calculated for each bin size; in this range; the statistical error was quantified as the corresponding standard deviation. For cells with more than one significant STC subunit, the STA was paired with every remaining STC subunit.

### Simulating filters recovered from responses to gaussian noise

We asked if the spatial structure of the subunits (STA and STC_K_) recovered from responses to Gaussian noise could be reconstructed by the weighted combination of two STAs obtained from responses to individually flashed dark and bright stimuli, STA_OFF_ and STA_ON_, respectively. We simulated the spatial structure (at peak amplitude) of the subunits of the receptive fields obtained with Gaussian noise by using a weighted combination of STA_ON_ and STA_OFF_.

The modeled STA subunit was defined as: model STA = a × STA_ON_ + b × STA_OFF_. The modeled STC subunit was defined as: model STC = c × STA_ON_ × STA_OFF_. The coefficients were optimized (fminsearch, Matlab) to minimize the sum of squared difference between the real and the modeled subunits (note we chose to use separate coefficients for the bright and dark STAs since using the same coefficient for both maps yielded poorer predictions). The pixel-wise difference between the real subunit and optimized model subunit formed the residual. The similarity between modeled and real subunits was measured using the signal to noise ratio, calculated as the logarithm of the ratio of power in the real subunit over the residual power.

## Results

The results were obtained from 23 adult female cats, ranging from 2.5–4.5 kg. We recorded from 121 cells in the PGN, 60 of which we held long enough for thorough study. Of these we were certain that 24 were binocular; 23 of the remaining cells were driven by the contralateral eye and 13 by the ipsilateral eye. In addition, we used 10 relay cells recorded from the same animals for control studies.

### Estimating receptive fields

Earlier studies described reticular receptive fields, qualitatively, as having diffuse shapes and having varying degrees of sensitivity to stimuli of opposite contrast (e.g., Sanderson, 1971; Dubin and Cleland, 1977; Uhlrich et al., 1991; Funke and Eysel, 1998). Our first goal was to provide a quantitative description of receptive fields in the PGN in order to specify the visual features that drive these cells. Thus we employed methods that had been successfully used to explore other stations in the early visual pathway including retina and cortex (Touryan et al., 2002; Rust et al., 2005; Fairhall et al., 2006; Schwartz et al., 2006). Specifically, we used STA and STC analysis of responses to dense noise to recover the first and second order features of the stimulus that neurons encode. As we will describe later, dense noise is an ideal stimulus for further types of analysis.

The STA reveals the first order patterns to which a neuron responds; see Schwartz et al. (2006) for review. The STA can provide a fine account of the receptive field if On and Off subregions are spatially separated, as for retina, LGN and simple cells in cortex. However, if there are regions in the receptive field where On and Off response overlap, then the influence of the weaker contrast is masked in the STA computed from responses to dense noise. At the extreme, equally strong On and Off responses are cancelled.

STC analysis recovers second order features of the stimulus that drive a cell. For example, it provides a means to explore sensitivity to On and Off stimuli presented to the same part of the receptive field. To perform the analysis, the STA is projected out from the spike-triggered stimulus ensemble, after which the axes of high and low variance (eigenvectors) in the covariance matrix are found using principal component analysis. Transforming the eigenvectors back to the image space reveals the second order spatiotemporal features that a given cell encodes (Schwartz et al., 2006). This approach has been successful in exploring complex cells in cortex, for example Rust et al. (2005). Taken together, the results of STA and STC analysis provide sets of linear subunits (also called spatiotemporal filters) that characterize the first and second order visual features that reticular cells encode.

**Figure 4:**
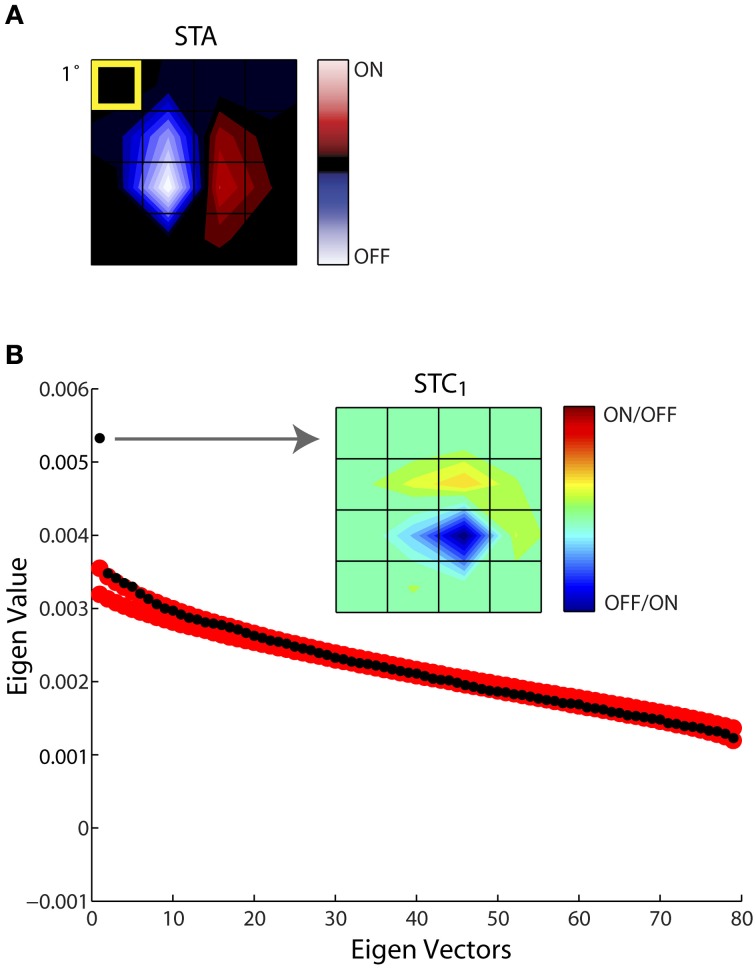
**Establishing the significance of the filters obtained with STC. (A)** STA recovered for a sample cell, shown as 4 × 4 window of the entire stimulus grid. **(B)** Principal component analysis of a (4 × 4 × 5) spike-triggered stimulus ensemble yielded 80 eigenvalues (black dots) plotted in descending order. Only one point fell above the upper or lower bounds of the 99% confidence interval determined from eigenvalues obtained from 1000 iterations of a nested bootstrap (red dots); the corresponding subunit is indicated by the arrow. A separate color code is used for maps obtained using STC since this method does not specify which subregions are On vs. Off. The eccentricity of the receptive field was 10.3°.

Figure 4 illustrates this process of estimating the subunits of the receptive field for an example cell. One spatiotemporal frame of the STA is shown in Figure 4A. In this and all remaining figures we display only the small window of the larger stimulus grid that contained the receptive field; in this case the STA had 80 dimensions (spatial, 4 × 4; temporal, 5). Subsequent STC analysis yielded a series of 79 eigenvectors, ranked according to their eigenvalues, black circles. The last (80th) eigenvalue, zero, is not shown since it was removed prior to the analysis; Figure 4B. We used a nested bootstrapping method to determine the significance of the eigenvectors (Schwartz et al., 2006). For this cell, only the first eigenvector was significant; that is, it lay beyond the confidence interval determined by the bootstrap, red circles, Figure 4B. The spatial filter corresponding to the significant eigenvector is displayed at right, indicated by the arrow. We refer to this filter as STC_1_; a second significant filter would be called STC_2_ and so on. Note we use a new color code for these subunits since STC analysis does not provide information about the absolute sign of preferred features.

Of the 60 cells whose responses to Gaussian noise we recorded, we recovered filters from both STA and STC analysis for 25 cells. The STC subunits were obtained with an average of 20–60 spikes per stimulus dimension. Obtaining even more spikes rarely seemed to improve the chances of recovering additional subunits. Also, substituting independent component analysis for principal component analysis did not increase the number of filters recovered (data not shown). Further, the presence or absence of significant STC subunits did not correlate with the changes in depth of anesthesia, as judged from the power spectrum of the EEG. Last, virtually all significant filters we extracted were excitatory rather than suppressive.

### Spatial structure of reticular receptive fields

By recovering the first and second order subunits of the receptive field, we were able to ask if similar patterns emerged across cells or if there were great heterogeneity in the population. We measured diversity in the receptive field by evaluating the relative magnitude of the On and Off subregions in the STA and STC subunits recovered from single cells. Our metric used the ratio between the absolute maxima of the On and Off subregions, setting the numerator to the highest peak and the denominator to the weaker one. A small value, near 1, indicated the presence of spatially separate On and Off peaks. We refer to subunits whose values for the “peak dominance” ratio was >2 as single peaked and those with values ≤2 as double peaked for ease of description. (Note that the relationship between overlapped subregions in the STA is “winner-take-all,” so a single peaked STA need not indicate an exclusive preference for stimuli of only one contrast.)

**Figure 5:**
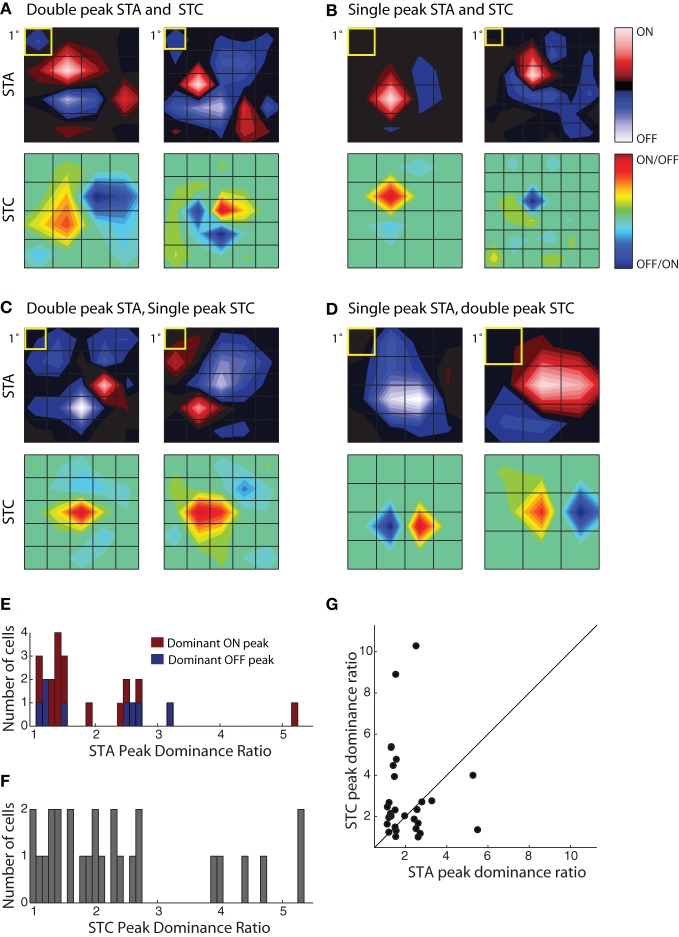
**Receptive field diversity in the PGN. (A–D**) Each panel illustrates spatial maps of subunits (STA, top and STC bottom) of the receptive field for two cells with similar receptive fields (yellow squares indicate 1°visual angle). The cells in the different panels are grouped according to the values of the peak dominance ratio for the STA and STC subunits. This ratio compares the relative strength of ON and OFF peaks, a value of one indicates subunits with two equally strong On and Off peaks and values greater than one indicate progressive differences between strengths of On or Off peaks. **(A)** Cells with double peaked STA and STC subunits; the peak dominance ratios for STAs, 1.49, 1.19 and STCs, 1.28, 1.24. The eccentricities were 10.6° (left) and 29.3° (right). **(B)** Cells whose STA and STC subunits were dominated by a single On or Off peak, peak dominance ratios for STAs, 5.2, 2.51 and STCs, 4, 10.28. The eccentricities were 8.3° (left) and 6.6° (right). **(C)** Cells with an STA with strong On and Off subregions but a single peaked STC; the peak dominance ratios for STAs are 1.2, 1.1 and for STCs, 2.68, 2.46. The eccentricities were 16.2° (left) and 14.2° (right). **(D)** Cells with a single peaked STA but double-peaked STC subunit; the peak dominance ratio for STAs are 2.60, 5.49 and for STCs, 1.01, 1.35. The eccentricities were 8.1° (left) and 4.1° (right). **(E)** Histogram of the peak dominance ratios for all STA subunits (*n* = 23); cells that prefer bright or dark stimuli are shaded red and blue, respectively. **(F)** Same as **(E)**, but for STC subunits, all cells are indicated by the same color, gray (*n* = 27); two points >6 were omitted from the plot to avoid compressing the abscissa. **(G)** Scatter plot of peak dominance ratio for pairs of subunits (STA and STC) obtained from single cells; cells with two STC subunits are represented twice.

There was a wide range in the relative strength of On and Off subregions in both STA and STC subunits, Figure 5. We first illustrate two cells that had a double peaked STA and STC_1_ in which the On and Off subregions were of roughly similar strength for both subunits; in this case the peak dominance ratios were near one, Figure 5A. Note, however, for each of these two cells, the shape of the STA and STC_1_ were different. Figure 5B depicts two cases for which the STA and STC had only a single peak. Even though the STAs resembled those of relay cells in the LGN, these reticular cells had overlapping dark and bright responses, as captured by filters recovered with STC analysis. In other instances, the relative weights of On and Off peaks in STAs were different from those in the filters recovered with STC analysis. Examples of cells with a double peaked STA, but single peaked STC_*K*_'s, are illustrated in Figure 5C while cells with reverse arrangement of subunits are illustrated in Figure 5D.

To assess the extent of spatial diversity of reticular receptive fields, we plotted a histogram of values for the peak dominance ratio for each STA and STC_*K*_ recovered for the population, Figure 5E. The plot of the STAs is color coded according to preference for stimulus contrast. It shows that strong responses to both bright and dark stimuli are common, Figure 5E. The plot also suggests that the relative weight of On and Off subregions varies continuously across the population. The distribution of peak dominance ratios is similar for subunits obtained with STC analysis, Figure 5F. Last, we compared the peak dominance of STA and STC subunits for the same cells, Figure 5G. Most points lie at some distance above or below the line of unity slope, further supporting the idea that neural receptive fields in the PGN are selective for many different combinations of visual features.

### Linear-non-linear models of the PGN

#### Components of the model

Quantitative maps of the spatiotemporal receptive fields in the retina and the LGN allow one to build simple computational models that predict neural responses reasonably well (Simoncelli et al., 2004; Carandini et al., 2005). But the complicated and diverse shapes of reticular receptive fields hinted that these simple models might not perform as well in the PGN. To determine how well subunits recovered from spike-triggered analyses help to predict neural responses in the PGN, we used simple LN models (Simoncelli et al., 2004; Carandini et al., 2005). The linear stage of the model was built using one or more filters (derived from STA or STC analysis) that were convolved with a time varying visual stimulus to generate a “filter output.” The non-linear stage comprised a static non-linearity, one for each subunit, which mapped the filter output to the strength of the corresponding neural response.

**Figure 6:**
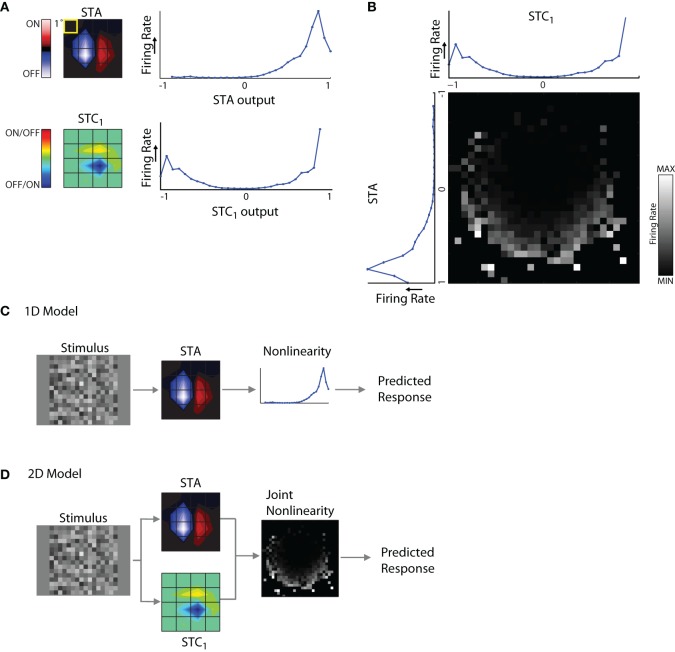
**1D and 2D LN models. (A)** Subunits recovered using STA and STC analysis with the corresponding response non-linearities for an example cell. **(B)** Joint response non-linearity, displayed as grayscale map, with the corresponding 1D non-linearities shown at the *left* and *top*; the brighter entries in the map indicate stronger neural responses. **(C)** 1D LN model; the stimulus is filtered by the STA subunit and passed through the non-linearity to generate a prediction of the neural response. **(D)** 2D LN model, same as for panel **(C)** except the stimulus is filtered by both the STA and STC_1_ subunit before passage through the joint non-linearity.

The components of the model are illustrated for a cell from which we recovered an STA and STC_1_ subunit, Figure 6A. A snapshot in time for each linear spatiotemporal filter (STA and STC_1_) is shown as a contour plot at left, next to its associated non-linearity. The non-linearities are curves plotted as firing rate against the filter output (see “Methods” for greater detail). One can think of the non-linearity as a lookup table that maps a given value of the filter output to a (mean) firing rate.

The non-linearities for the STA and STC subunits have different shapes. For the STA, stimuli with the same polarity as the filter produced positive outputs and led to elevated spike rates; further, firing rate grew with the magnitude of the filter output. By contrast, filter outputs for stimuli that had the opposite polarity produced negative values that were not associated with notable changes in firing rate. Thus the shape of the non-linearity for the STA subunit was one-sided, or (approximately) half-wave rectified. The shape of the non-linearity associated with the STC subunit was qualitatively different; it was U-shaped. This is because both positive and negative values of the filter output were associated with elevated firing rates. In other words, firing rate grew larger as the absolute value of the filter output increased. For similar examples in the literature, see Rust et al. (2005), Touryan et al. (2005) and see Schwartz et al. (2006) for review.

These non-linearities were generated separately for each filter. However, interactions between subunits have the potential to influence neural responses. Such mutual influence can be estimated by constructing a joint non-linearity, Figure 6B. Here, filter outputs for STA and STC_1_ are plotted alongside a square grid whose entries represent firing rates evoked by the coincident activation of both subunits as they are variously engaged by the stimulus.

It was necessary to estimate the joint non-linearity separately for each model because interactions between the STA and STC subunits were not merely additive or multiplicative. In other words, if the joint non-linearity simply resulted from the point-wise products of the two individual non-linearities, then the stronger entries in the grid would have formed rectangular contours. However, these stronger entries assumed curved patterns, see Rust et al. (2005).

#### 1D and 2D models

Using these components, we were able to build two types of LN models to explore the relative contributions of each different subunit of the receptive field. These were 1D models that included only the STA, Figure 6D, and 2D models that also incorporated subunits derived from STC analysis, Figure 6D. For cells with two or more significant STC subunits, we made pair-wise assessments between each one and the STA. This was because it is difficult to collect enough data to estimate the joint non-linearity for more than two subunits at a time because of the very high number of filter combinations.

#### Assessing the performance of the models and exploring interactions between subunits using explained variance

**Figure 7:**
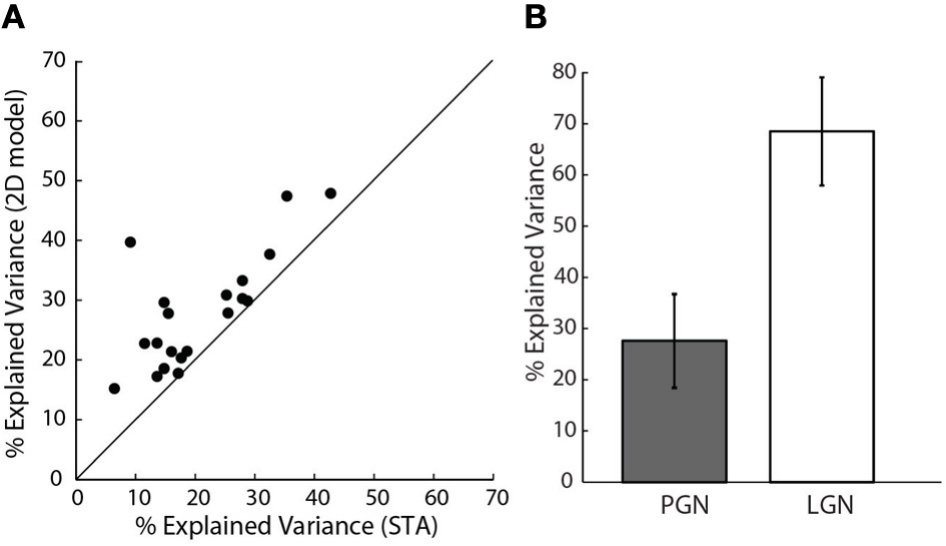
**Assessing model performance using explained variance. (A)** Explained variance for the 2D vs. 1D model, all the points lie above the line of unity slope. (*n* = 20). **(B)** Comparison of explained variance for 1D models of cells in the PGN (*n* = 20), shaded, and a randomly selected sample of cells in LGN (*n* = 10), unshaded.

How well did these models predict neural responses? Because the models were fitted using only half of the data recorded for each cell, we were able to test the performance using the remaining data, cross-validation (Hastie et al., 2001). We first compared the performance of the 2D to the 1D models, Figure 7A. The assessments were based on explained variance. This quantity is the amount of variance in the stimulus-driven neural response (i.e., spike rate), that the model predicts. The performance of the more elaborate models was always best. This is seen in Figure 7A, where all points fell above the line of unity slope in a plot of explained variance for the 1D vs. 2D models. Even the best 2D models, however, predicted only about 30% of the neural response; that is, they improved average values (21%) for the 1D models by approximately 50%. It is important to mention that these data exclude cells for which additional STC subunits were not significant, based on the bootstrap; for these cases, the 2D models were not better than those made with the STA alone.

Our initial calculations of explained variance used a method developed by others (Haefner and Cumming, 2009). One might fear that the low values we obtained resulted from an error in our implementation of these methods. Hence, we also used a different method that quantified the amount of stimulus-related signal power, see “Methods” (Sahani and Linden, 2003). This metric gave similar values; 22% mean explained variance for the 1D model and 30.6% for the 2D model.

#### Controls using data from the LGN

One might also wonder if our values of explained variance were low because of a problem unique to our preparation. To address this concern we compared predictions of 1D models for reticular cells to those made for relay cells in the LGN that we recorded in the same animals. We used only 1D models since we recovered only STAs from relay cells. The explained variance for relay cells ranged between 60 and 70%, Figure 7B, as has been reported elsewhere (Mante et al., 2008). Thus, the low values of explained variance we obtained for reticular cells seemed to reflect intrinsic properties of the PGN.

### Exploring interactions between subunits using measures of mutual information

Does each subunit make an independent contribution to the neural response or might their interactions reveal redundancy or synergy? To address this question, we explored interactions between subunits using information theory. We assessed the amount of encoded information about single subunits vs. jointly about multiple subunits by estimating the mutual information between the component filters and the neural response. This was done by calculating the Kullback–Leibler divergence between the distribution of filter outputs for the entire length of the stimulus period (the prior distribution) and the distribution of filter outputs just before each spike (the spike conditional distribution), using methods developed previously (Aguera Y Arcas et al., 2003; Fairhall et al., 2006). A difference between the two distributions indicates that the filtered stimulus provides information about the neural spike train.

**Figure 8:**
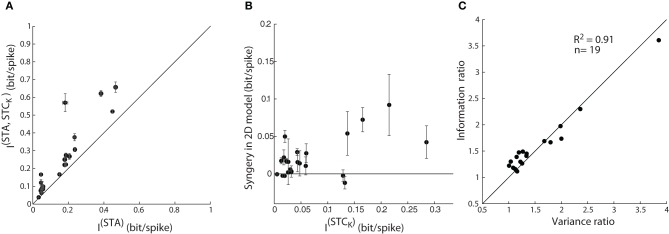
**Assessing model performance by using information theory. (A)** Comparison of mutual information estimated from one vs. jointly by two subunits; as in Figure 7A. The error bars depict the standard deviation of the information value (circles) computed for different bin sizes used. **(B)** A plot of synergy (the amount of additional information encoded by the pair of subunits vs. the summed information encoded independently by the two subunits) against information encoded by a single STC subunit. **(C)** Ratio of mutual information by a single vs. pair of subunits plotted against the corresponding ratio for explained variance accounted by 1D vs. 2D model; all points lie near the diagonal.

The initial step in the analysis was to address the problem of finite sampling of a continuous signal, which we did by resampling the filter output at different bin sizes, as described by Fairhall et al. (2006) (see “Methods”). Then, we calculated the information from the STA alone, I^(STA)^, as well as the joint information available from the pairs of two subunits, the STA and a (significant) STC subunit, I^(STA, STC_K_)^. The mutual information (between stimulus and response) obtained from the paired subunits I^(STA, STC_K_)^, was significantly greater than that available from the STA alone, I^(STA)^, Figure 8A. So far, these results parallel those for explained variance in Figure 7A and seem straightforward.

Next, we assessed the possibility of synergistic or redundant influences of one subunit on another. Thus, we determined if the amount of joint information, I^(STA, STC_K_)^, exceeded that from a simple combination of I^(STA)^ and I^(STC_K_)^. Synergy was defined by values >0 in an index that subtracts (I^(STA)^ + I^(STC_K_)^) from I^(STA, STC_K_)^, as outlined in previous work (Fairhall et al., 2006). Independent contributions would equal zero whereas redundant interactions would score <0. The plot for the population, Figure 8B, shows that there are synergistic interactions between subunits recovered from most cells and only rare cases of redundancy.

Last, the information theoretic analysis served a second function, as a control for our methods of estimating the performance of the 1D and 2D models. Specifically, we compared values of explained variance obtained earlier with those for mutual information. A ratio of the explained variance for the 2D vs. 1D models is plotted against the ratio of the I^(STA, STC_K_)^vs. I^(STA)^ for each reticular cell, Figure 8C. Most points fell along the unity line (*R*^2^ = 0.91), indicating that both measures are equally good at assessing the performance of the LN models we made.

### Can STAs mapped with sparse noise (individual bright or dark stimuli) predict STA and STC subunits obtained using dense (gaussian) noise?

So far, we have discussed how we recovered subunits of reticular receptive fields, how we assessed their predictive power and estimated the amounts of information they encode. However, we have not addressed the question of how these subunits might be formed. In other words, we wondered how the subunits recovered from responses to Gaussian noise might reflect the On and Off inputs that relay cells supply. Recall that our previous analyses revealed a wide range in the relative strengths of On and Off contributions to filters recovered using STA and STC analysis, Figure 5. For example, some cells seemed to respond well to On or to Off stimuli while others strongly preferred stimuli of just one polarity.

The simplest explanation for the shapes and signs of the various filters is that these were built from a linear combination of On and Off subregions whose peaks were spatially displaced to varying degrees. For many of the cells we mapped with Gaussian noise, we also collected companion datasets with sparse noise, individually flashed bright and dark pixels. The sparse noise allowed us to recover separate STAs for bright and dark responses; we refer to these specifically as (STA_ON_) and (STA_OFF_), respectively (we continue to use the term STA, without subscript, to refer to maps made with Gaussian noise). With the results of the sparse noise mapping, it was a simple matter to determine if the subunits (STA and STC_K_) recovered using Gaussian noise could be simulated by the weighted sum or product of the maps acquired with sparse noise, STA_ON_ and STA_OFF_.

**Figure 9:**
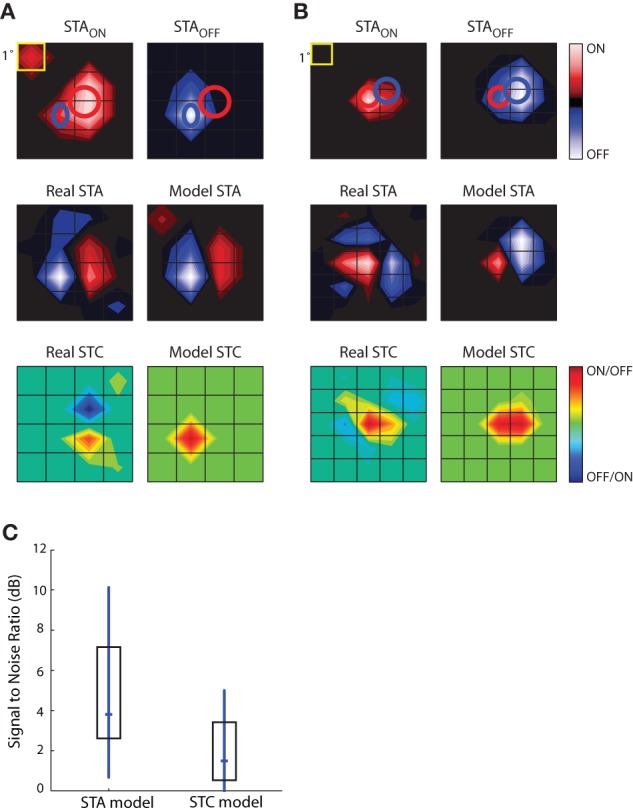
**Simulations to explore the construction of receptive fields.** STAs for sparse noise are used to predict the STA and STC subunits recovered from responses to dense (Gaussian) noise. **(A)** Top, STAs made for bright (STA_ON_, left) and dark (STA_OFF_, right) spots, ellipses fit to the peak of each subregion are shown in both maps. Middle, STA recovered from response to dense noise (left) next to a modeled STA (right) constructed from the weighted sum of the STA_ON_ and STA_OFF_ shown above. Bottom, STC recovered from dense (Gaussian) noise (left) and the modeled STC (right) that was made using the weighted product of the STA_ON_ and STA_OFF_. **(B)** Same as **(A)**, but for a different cell. **(C)** The match between the modeled STA and real STA is greater than that between the modeled STC and the actual STC, as quantified by plots of the signal to noise ratio; median (horizontal dash) and interquartile range (box).

The results of this analysis are depicted for two different cells in Figures 9A,B. The top row shows the STA_ON_ and STA_OFF_ maps; overlaid red and blue ellipses that were fit to the peaks illustrate the spatial offset between On and Off subregions. The middle rows compare the actual STAs obtained with Gaussian noise to those modeled by taking the weighted sum of those acquired with sparse noise (STA_ON_ and STA_OFF_). Specifically, the model was STA = *a* × STA_ON_ + *b* × STA_OFF_, where the coefficients, *a* and *b*, were optimized to reduce the mean square error between the model STA and the actual STA made from responses to Gaussian noise. The main features of the modeled STAs, prominent On and Off peaks and their relative positions, were similar to the actual STAs acquired using Gaussian noise, suggesting that the maps recovered from Gaussian noise approximate the weighted sum of On and Off inputs. This rough match between the modeled and real STAs was seen for all cells we were able to test, regardless of the relative strength or spatial displacement between On and Off subregions. The sparse noise analysis also allowed us to assess the degree of spatial and temporal overlap between STA_ON_ and STA_OFF_, which we did by calculating the normalized dot product of STA_ON_ and STA_OFF_. For our sample of eight cells, the spatial overlap was 64.26 ± 23.42%, mean ± standard deviation. The peaks of both STA_ON_ and STA_OFF_ occurred during the same time interval.

The subunits recovered using STC analysis of responses to Gaussian noise reflect sensitivity to On and Off inputs. To model these subunits, we used a multiplicative model. Specifically, we modeled each STC as the weighted product of the STA_ON_ and STA_OFF_ acquired using sparse noise(*c* × STA_ON_ × STA_OFF_). Non-zero values of the resulting product indicate the regions where On and Off subregions overlap. These models failed to reproduce the spatial structure of the STCs. At best, the filters produced by this simple operation captured one peak of the actual STC; even then, the modeled peak was often displaced from the actual. This result suggests that reticular cells do not pool their inputs in a simple fashion.

The next step in this analysis was to quantify the degree to which the real and modeled filters matched, which we did by calculating the signal-to-noise ratio. This metric is the logarithm of the ratio of power in the real subunit over the power of the error, with the latter defined as pixel-wise difference between the real and modeled subunit. As expected from visual inspection of the maps displayed in Figures 9A,B, the performance of the modeled STA was better than that of the modeled STC; Figure 9C. Thus, the weighted summation of On and Off inputs is able to explain overall spatial structure of the linear filters (STAs) recovered from response to a rich stimulus. On the contrary, a simple product of On and Off maps cannot reliably estimate filters generated by non-linear interactions (STCs) between bright and dark stimuli.

## Discussion

The first feedback loop in the early visual pathway is formed by connections between the LGN and PGN. The PGN has long been thought to provide a non-selective form of gain control to relay cells (Levick et al., 1972; Dubin and Cleland, 1977) regulated by attention (McAlonan et al., 2006, 2008). We explored stimulus specificity in the PGN by identifying visual features that reticular neurons encode, using recordings from the LGN as controls. We estimated receptive fields from responses to various types of noise, using STA and STC analyses (Schwartz et al., 2006) that we adapted for the statistics of bursting neurons. For almost half the sample, we were able to recover both first (STA) and higher order subunits (STC_K_) of the receptive field from responses to dense noise (Gaussian checkerboards). These STAs could be explained by the weighted sum of maps obtained using individual dark and bright squares but STC subunits could not be approximated by simple combinations of On and Off maps, suggesting that complex mechanisms underlie selectivity for higher order features. Further, we built LN models to assess the predictive power of the receptive fields. For cells in the PGN, the performance of these models almost always fell below that achieved for relay cells, although including more than one filter improved predictions. Moreover, information theoretic analyses revealed that multiple subunits interact to encode information synergistically.

In aggregate, our analyses show that neurons in the PGN are sensitive to both first and second order features of the stimulus and, it stands to reason, provide inhibition that is highly selective for visual pattern. Given that individual reticular cells receive convergent inputs from LGN, their responses might vary dynamically as the stimulus recruits different subsets of presynaptic cells. Further, our results are consistent with studies of the broader thalamic reticular nucleus; neurons in the somatosensory region are tuned for direction (Hartings et al., 2000) and cells in the auditory division prefer rich acoustic patterns (Simm et al., 1990). This emerging view of the thalamic reticular nucleus as being sensitive to complex patterns is especially interesting given recent work that suggests that bottom–up mechanisms of human attention are guided by second order features of the visual scene (Frey et al., 2007).

### Adapting spike-triggered methods of analysis for neurons that fire long bursts

Most spike-triggered methods involve the implicit assumption that each impulse is independent from the next (Pillow and Simoncelli, 2003; Schwartz et al., 2006) so that spike trains are free of autocorrelations. However, work *in vitro* (Huguenard and Prince, 1992) and *in vivo* (Contreras et al., 1993) suggests that this assumption is violated for reticular cells, which fire long bursts. Methods we developed showed that a common stimulus initiates all spikes in burst.

Further, we observed that the spatial structure of receptive fields constructed from either the cardinal spikes of bursts or from tonic action potentials were similar. Thus, we used a mixture of both types of spikes for further analysis. This observation recalls work in LGN that used white noise to compare receptive fields formed with tonic spikes vs. bursts; both maps had the same shapes, with subregions in the bursts-triggered averages slightly stronger (Alitto et al., 2005). Stimuli with strong spatial and temporal correlations (gratings, naturalistic patterns), however, seem to evoke bursts far more effectively than noise (Alitto et al., 2005; Denning and Reinagel, 2005; Wang et al., 2007; Niell and Stryker, 2010).

### Neurons in the PGN encode first and higher order features of the stimulus

Earlier studies reported that various proportions of neurons in the PGN respond to bright and dark stimuli (Sanderson, 1971; Dubin and Cleland, 1977; So and Shapley, 1981; Uhlrich et al., 1991; Funke and Eysel, 1998) and that receptive fields were often diffuse (Sanderson, 1971; Uhlrich et al., 1991; Funke and Eysel, 1998). We wished to provide a quantitative view of the visual features that reticular neurons select.

Using STA and STC analysis of responses to dense noise, we recovered the first and second order subunits that contribute to the receptive fields in the PGN. The relative strengths of On and Off subregions in the STAs varied widely as noted in earlier qualitative descriptions (Dubin and Cleland, 1977; Uhlrich et al., 1991; Funke and Eysel, 1998). At one extreme, the STA comprised equally strong On and Off subfields, at the other end of spectrum, a single contrast dominated the filter. STC analysis identified additional filters for ~40% of our sample. Further, the shapes of the STAs and STCs did not co-vary. For example, cells whose STA had prominent On and Off subregions might have a STC subunit dominated by a single-contrast, and vice versa. Thus, receptive field structure in the PGN is not stereotyped, as in the LGN; different reticular cells are selective for different specific visual features.

The success or failure to recover multiple filters did not depend exclusively on the number of spikes recorded, the shape of the STA, or depth of anesthesia. Nor were subunits missed because we used principal component analysis (for which the STA and STC_K_ are orthogonal) to assess the STC; independent component analysis [for which filters need not be orthogonal, (Saleem et al., 2008)], yielded comparable results. However, since the STA was removed before estimating the STC, we would have lost the latter if both filters were similar. Further we would not have recovered subunits that represent >2nd order statistics of the stimulus. Last, we expected that inhibitory subunits would be common since connections intrinsic to the PGN are GABAergic, but this was not the case. Others have suggested that STC analysis might be insensitive to suppression that is distributed across multiple subunits (Touryan et al., 2005).

### Contribution of on and off maps acquired separately with sparse noise to filters recovered using dense noise

We asked if the spatial diversity in the filters we recovered reflected variously complex mechanisms of response, or simple interactions between On and Off subregions. Hence we determined if the STAs acquired with dense noise could be reproduced by summing weighted maps made using flashed dark and bright spots. This method provided a fair approximation of the STAs recovered from the dense stimulus. By contrast, filters recovered from STC were poorly simulated by taking the product of these On and Off maps. Thus, these higher order subunits seem to derive from strongly non-linear interactions.

### Potential circuits that build the receptive field

What underlying circuits might generate the receptive fields we mapped? Some STAs had adjacent On and Off subregions, reminiscent of the simple receptive fields (On and Off subregions lie side by side) characteristic of the cortical cells that project to thalamus (Hirsch et al., 1998b). Nonetheless, this observation does not imply a role for corticothalamic feedback. As for simple cells (Hubel and Wiesel, 1962; Reid and Alonso, 1995; Hirsch and Martinez, 2006), the spatial structure of these STAs can be explained by input from relay cells. This idea is supported by the finding that shape of the STA could be simulated by the weighted sum of separate On and Off maps. The structure of the STC subunits could not be described by the product of On and Off maps, however, perhaps reflecting non-linear contributions from the PGN.

Additional, complementary, lines of evidence suggest that the responses we recorded were driven by feed-forward input. The STAs and STCs had similar latencies and time-courses, indicating a common origin [cortical contributions arrive at longer delay (Briggs and Usrey, 2008)]. Further, removing or silencing cortex has modest influence on the PGN (Sanderson, 1971; Xue et al., 1988; Jones and Sillito, 1994). Third, synapses from relay cells onto reticular cells are large and proximal (Montero and Singer, 1984; Cucchiaro et al., 1991; Bickford et al., 2008) whereas corticothalamic boutons are small and may favor distal sites (Cucchiaro et al., 1991; Bickford et al., 2008). Thus, it seems likely that the LGN drives the PGN whereas the cortex serves a modulatory role (McAlonan et al., 2008).

### Models of the reticular receptive field, predictive power and ability to encode information

Simple LN models successfully predict responses recorded from retina (Pillow et al., 2005; Zaghloul et al., 2005), LGN (Mante et al., 2008; Wang et al., 2011), and simple cells in V1 (Carandini et al., 2005). However, for cortical neurons with non-linear responses, computational models fare less well e.g., David and Gallant (2005). Such was the case for the PGN. Simple models using the first order filter (STA) alone accounted for only ~20% of responses to dense noise, compared to ~70% for controls in the LGN. Adding additional subunits (STC_k_) to the models always bettered performance but rarely brought the total explained variance near levels, achieved for relay cells.

Factors likely to limit the success of LN models of the PGN include unique membrane properties of reticular neurons (Huguenard and Prince, 1992), adaptive mechanisms for luminance and contrast (Mante et al., 2005, 2008), and the complexity of local circuits (Montero and Singer, 1984; Cucchiaro et al., 1991; Uhlrich et al., 1991; Wang et al., 2001; Landisman et al., 2002; Bickford et al., 2008).

Last, we asked if the different subunits conveyed information independently. We compared the amount of information encoded by pairs of filters (STA and STC_K_) to the amount estimated from either subunit alone. The pairs often encoded significantly more information than predicted from the sum of that encoded by single filters, suggesting that subunits interact synergistically. All told, understanding the function of reticular neurons seems no less challenging than exploring the visual cortex—the ultimate destination for relay cells that innervate and receive feedback from the PGN.

## Author contributions

Vishal Vaingankar and Cristina Soto-Sanchez performed the experiments with help from Judith A. Hirsch and Xin Wang. Vishal Vaingankar, Cristina Soto-Sanchez, and Friedrich T. Sommer contributed to most analyses with additional assistance from Xin Wang. Vishal Vaingankar, and Judith A. Hirsch wrote the manuscript. Vishal Vaingankar prepared the figures.

### Conflict of interest statement

The authors declare that the research was conducted in the absence of any commercial or financial relationships that could be construed as a potential conflict of interest.
